# Myocardial Work Index in Professional Football Players: A Novel Method for Assessment of Cardiac Adaptation

**DOI:** 10.3390/jcm12093059

**Published:** 2023-04-23

**Authors:** Elena Refoyo, Jesús Troya, Ana de la Fuente, Almudena Beltrán, Oscar Luis Celada, Leonel Díaz-González, Roberto Pedrero-Tomé, Manuel García-Yébenes, Jose María Villalón

**Affiliations:** 1 Department of Cardiology, Clínica Universidad de Navarra, 28027 Madrid, Spain; 2 Cardiac Imaging Unit, Department of Cardiology, Hospital Universitario la PAZ, IdiPAZ, 28046 Madrid, Spain; 3 Department of Internal Medicine, Hospital Universitario Infanta Leonor, 28031 Madrid, Spain; 4 Department of Internal Medicine, Clínica Universidad de Navarra, 28027 Madrid, Spain; 5 Club Atlético de Madrid—Medical Services, 28221 Madrid, Spain; 6 Infanta Leonor Hospital Research and Innovation Foundation, 28031 Madrid, Spain; 7 EPINUT Research Group, Faculty of Medicine, Complutense University of Madrid, 28040 Madrid, Spain

**Keywords:** echocardiography, myocardial work, athlete’s heart, two-dimensional speckle tracking

## Abstract

Background: The global myocardial work index (GWI), a novel, valid, and non-invasive method based on speckle-tracking echocardiography, could provide value for calculating left ventricular (LV) function and energy consumption in athletes. Materials and Methods: We prospectively analyzed a single-center cohort of Spanish First-Division football players who attended a pre-participation screening program from June 2020 to June 2021, compared to a control group. All the individuals underwent an electrocardiogram and echocardiography, including two-dimensional speckle tracking and 4D-echo. The study aimed to evaluate the feasibility of myocardial work in professional football players and its correlations with other echocardiographic parameters. Results: The study population comprised 97 individuals (49 professional players and 48 controls). The mean age was 30.48 ± 7.20 years old. The professional football players had significantly higher values of LVEDV (*p* < 0.001), LVESV (*p* < 0.001), LV-mass index (*p* = 0.011), PWTd (*p* = 0.023), and EA (*p* < 0.001) compared with the control group. In addition, the professional players had lower GCW (*p* = 0.003) and a tendency to show lower GWI values (*p* < 0.001). These findings could suggest that professional football players have more remodeling and less MW, related to their adaptation to intensive training. Significant differences in GLS (*p* = 0.01) and GWE (*p* = 0.04) were observed as a function of the septal thickness of the athletes. Irrespective of the MW variable, the parameters with better correlations across all the populations were SBP, DBP, and GLS. Conclusions: The GWI is a novel index to assess cardiac performance, with less load dependency than strain measurements. Future GWI analyses are warranted to understand myocardial deformation and other pathological differential diagnoses.

## 1. Introduction

Physical exercise and sports have demonstrated cardiovascular benefits [1]. Participation in regular intensive sports requires a five- to six-fold increase in cardiac output, which induces structural and functional cardiac adaptations, defined as athlete´s heart. These changes include chamber dilatation, ventricular hypertrophy, enhanced diastolic filling, and changes in autonomic function [2]. In most cases, the adaptations of athletes’ hearts manifest with high-intensity-exercise stimuli, frequently performed, of prolonged duration, and with the involvement of large muscle masses [3]. In clinical practice, it can be challenging to distinguish between patients with early cardiac disease and those with exercise-adaptive changes, which often coexist in the so-called gray zones [3,4].

Common cardiovascular diseases observed in athletes can be suspected based on an electrocardiogram (ECG), in which is a cost-effective test [5]. However, the echocardiogram has been proposed as a valuable tool in diagnosing not only different diseases in athletes, such as cardiomyopathies, valvulopathies, congenital heart diseases, myocarditis/pericarditis, and cardiac fatigue, but also in assessing the physiological adaptations to intense physical activity [6,7]. These physiological changes vary according to the type and intensity of the sport practiced. However, it is accepted that increased left ventricular (LV) end-diastolic volume (EDV), eccentric hypertrophy, and augmented stroke volume associated with electrical changes, such as sinus bradycardia, are standard features of the athlete’s heart [8,9].

Speckle-tracking echocardiography (STE) is a relatively recent echocardiographic technique of deformation imaging that has provided new insights into the characterization of athletes’ myocardial properties [10]. It can detect subclinical ventricular systolic function in early-stage cardiac disease when LVEF is normal. In previous reports, 2D-derived global longitudinal strain (GLS) failed to show significant changes in athletes; some studies reported lower GLS values in athletes with endurance training compared with strength training and healthy controls. Strain measurements (preload, afterload, LV mass, and sinus bradycardia) could explain these differences [11].

Myocardial work (MW) has been established recently as a new LV systolic-function index. It considers afterload based on strain and non-invasive LV pressure as a surrogate measure for overall myocardial consumption [12,13]. The increased afterload may impair strain in different physiologic and pathologic conditions, with preserved or increased MW indices. This aspect may be relevant for athletes who exhibit variable blood pressure and loading conditions at different seasonal stages [14].

Although these cardiac changes induced in athletes have been described in other sports, such as swimming or cycling, data regarding professional football players are scarce. Therefore, this study aimed to evaluate the feasibility of analyzing myocardial work in professional football players and its correlations with other echocardiographic parameters.

## 2. Materials and Methods

### 2.1. Population and Study Design

We prospectively enrolled a single-center cohort of Spanish First-Division football players who had followed an intensive training schedule of more than 15 h a week for over five years. The control group was recruited from healthy hospital staff who did not undertake regular physical exercise. Both groups were in the same age range and were matched by body-mass index (BMI), and the recruitment period lasted from June 2020 to June 2021.

Professional football players were selected after performing FIFA-mandated pre-participation health screening before competitions. As a result, all players presented similar training methods, emphasizing effort directed toward improving power and speed rather than endurance, despite some variability based on the players’ positions.

Selection criteria for both groups included: (a) exclusion of metabolic diseases, including hypertension; (b) normal 12-lead ECG; (c) LV ejection fraction > 55%; (d) normal wall-motion-score index, and exclusion of myocardiopathy; (e) exclusion of genetic or congenital heart disease; and (f) exclusion of valvular or coronary pathology In addition, for the control group, (g) lack of regular exercise (cut-off pf less than 3 h a week) was applied.

Professionals and controls underwent an ECG, standard echocardiography, and a complete clinical and laboratory evaluation. In addition, clinical data (risk factors, comorbidities, medical therapies, and physical exercise) were collected from medical records and entered with echocardiographic variables into an online electronic database, Redcap [15].

### 2.2. Echocardiographic Methods

Transthoracic echocardiography was performed using a commercially available ultrasound system (Vivid™ E95 4D ultrasound system, GE Healthcare, Milwaukee, WI, USA) with 3.5-MHz M5Sc 2D and a 3D volumetric transducer (4 V) for real-time echocardiographic dataset acquisition of the ventricles. Two operators conducted 2D and 3D echocardiography on the same machine to avoid multiple-observer and inter-device variability and discrepancies in the techniques. Ultrasonic recordings and measurements were processed according to the European Association of Cardiovascular Imaging recommendations [16].

Left-ventricular-internal-dimension diastole (LVIDd), diastolic interventricular septal thickness (IVSTd), and diastolic posterior-wall thickness (PWTd) were obtained in the parasternal long-axis section of LV. Relative wall thickness (RWT) was computed using the ratio (IVSTd + PWTd)/LVIDd). The LV end-diastolic volume (LVEDV), LV end-systolic volume (LVESV), and LV ejection fraction (EF) were calculated using Biplane Simpson’s method. Valvular regurgitation was quantified from color Doppler imaging and categorized as absent, minimal (within normal limits), or mild using the vena contracta method.

### 2.3. Two-Dimensional Speckle-Tracking Echocardiography (2DSTE)

Quantification of 2D strain was performed using commercially available software (EchoPAC™workstation CentricityTM Cardio Workflow, version 6.0 SP6, GE Healthcare, EEUU). The analysis was performed in all three apical views (LV four-, two-, and three-chamber). The operator assessed the tracking quality and scores were compiled by the software with an automated function in the region of interest, which was adjusted by correcting the endocardial border or width if deemed necessary.

Aortic valve closure was identified using the automated function from the apical long-axis view. The GLS was calculated by averaging local strains of all 16 segments and expressed as bull’s-eye. If the software did not correctly identify the LV-wall movement without assigning a strain value to the LV segment (poor tracking), the operator repeated the process, readjusted the endocardial tracing, or changed software parameters, such as region of interest (ROI) width and smoothing until a better score was achieved.

### 2.4. Myocardial Work

**Figure 1:**
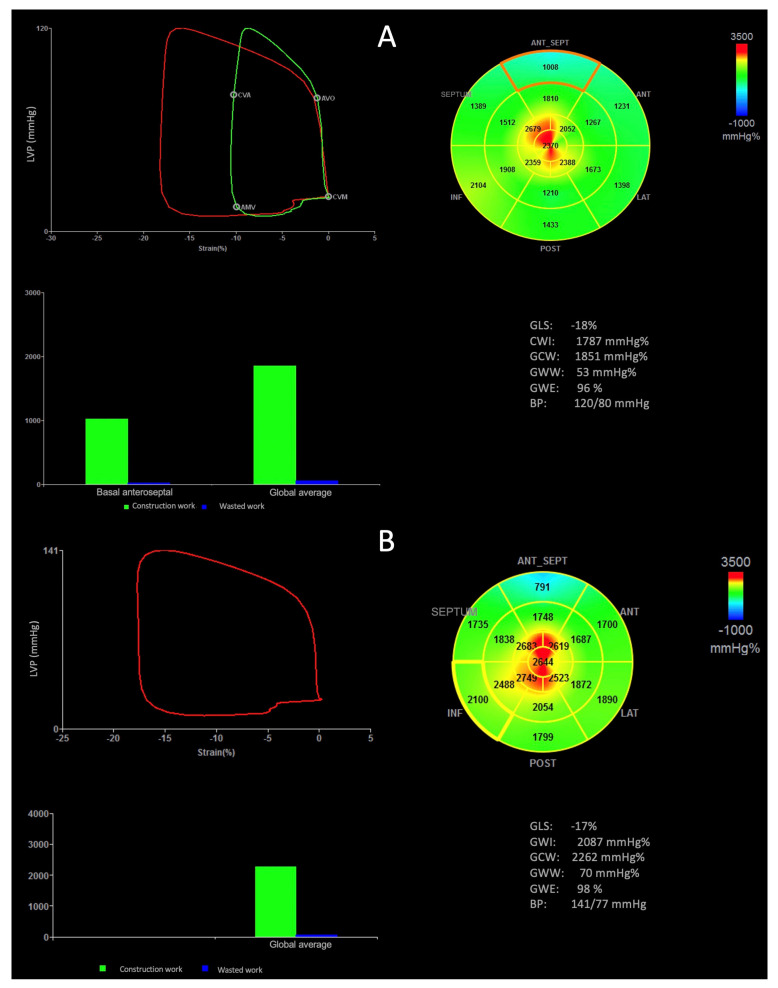
Measurement of myocardial work parameters by 2D echocardiography of the athlete group. (**A**) LV pressure–strain loop; bull’s eye of GWI; bar graph representing GCW and GWW; and results from myocardial work analysis on a healthy patient. The myocardial work bull’s eye shows areas of negative work in blue, green indicates normal values, and red shows high-work areas. (**B**) Fibrosis and reduced WI (38%) in the corresponding segment in a 24-year-old black male player diagnosed with anteroseptal hypertrophy.

Global myocardial work index (GWI) was calculated using a specific software with a combination of LV strain and a non-invasively estimated LV pressure with systolic cuff pressure. Peak systolic LV pressure was assumed to be equal to the peak arterial pressure, measured immediately before the echocardiographic study. The software constructed a non-invasive LV-pressure curve, which was adjusted according to the duration of isovolumic and ejection phases defined by valvular-timing events. The area within the curve was an index of MW. An additional set of indices was used: global constructive work (GCW), defined as myocardial work performed during segmental shortening; and global wasted work (GWW), defined as work performed by the LV that does not contribute to LV ejection, characterized by the lengthening of myocytes during systole, with addition of shortening during the isovolumic relaxation phase; and global myocardial work efficiency (GWE), calculated as constructive MW/(constructive MW + wasted MW) (0–100%) (Figure 1).

The proposed ranges for non-invasive MW in the NORRE (normal reference ranges for echocardiography) study were used as normality-reference values [17].

### 2.5. 3D Echocardiography

Beyond routine echocardiographic protocol, electrocardiographically gated full-volume 3D data sets, reconstructed from four or six cardiac cycles optimized for the LV, were obtained from apical view, with a minimum volume rate of 25 volumes/sec, for offline analysis. The quality of images was verified to avoid “stitching” and “dropout” artifacts in the 3D data. Further measurements were performed on a separate workstation using dedicated software.

### 2.6. Statistical Analysis

The normality of the distribution of continuous variables was tested using the Kolmogorov–Smirnov test. Continuous variables were expressed as means and standard deviation (SD). Parametric (Student’s *t*-test and Mann–Whitney U) and nonparametric (ANOVA and Kruskal–Wallis) tests were applied to contrast means. Correlations between the continuous variables of MW (GWI, GCW, GWW, and GWE), standard echocardiography, and advanced echocardiography were calculated using Pearson’s or Spearman’s correlation coefficient, as appropriate. Chi-square tests were used to evaluate the association between valvular regurgitations and the number of alterations as a function of amateur- or professional-player status and field position. Finally, binary logistic regression analyses were performed, in which the dichotomous dependent variables were the presence of valvular regurgitation and more than one alteration in the subsample of professional players. All statistical analyses were carried out using SPSS version 25.

The local ethics committee at Clínica Universitaria de Navarra approved the protocol, code PI 2019.77. All participants provided written informed consent.

## 3. Results

The study population comprised 97 individuals (49 professional football players and 48 controls). The demographic data, standard echocardiogram, left-ventricular myocardial work, and strain parameters of the professional football players and controls are summarized in Table 1.

In the standard echocardiographic measurements, the professional football players presented higher values for LV (LVEDV (*p* < 0.001), LVESV (*p* < 0.001)), mass index (*p* = 0.011), and PWTd (*p* = 0.023). At the same time, the LVEF values showed a minimal reduction without statistically significant differences. No regional wall-motion abnormalities were identified. Concerning the trans-mitral Doppler index, the professional players showed higher mitral E velocities and EA ratios (*p* < 0.001), as well as lower values for lateral E/E’ (*p* = 0.008).

The professional footballs presented higher right ventricular (RV) volumes in the advanced echocardiographic measurements, while the RV functionality did not show statistically significant differences. A 2DSTE analysis showed a mild reduction in LV GLS (*p* = 0.030) in the professional players, who had lower GCW (*p* = 0.003) than the control group and a tendency to lower values for GWI. The GWE did not show significant differences between the two groups.

Using normality-reference values of myocardial work [17] in a range of 20–40 years, the percentage of professional football players with lower values of GWI, GCW, GWW, and GWE were 44.7%, 73.7%, 94.7%, 10.5%, respecitvley, compared to 29%, 41.9%, 71%, and 22.6%, respectively, in the control group.

There were no significant differences between the demographic and echocardiographic characteristics according to the positions of the players on the field (Supplementary Table S1). Possible differences in this set of variables were analyzed as a function of the number of abnormalities in the group of professional football players (Supplementary Table S2). In this group, the subjects with the highest numbers of reported alterations were older (*p* = 0.004) and presented higher RVEDV (*p* = 0.007) and RVESV (*p* = 0.010). Regarding the MW, there was a certain tendency towardhigher GCW and GWW in the subjects with greater alterations.

It should be noted that, given the sample sizes of both groups (controls and professional footballers), there were numerous variables with *p*-values close to significance (*p* < 0.05). In these specific cases, extreme caution should be exercised, as we cannot establish a trend until we observe how these variables behave in larger samples.

**Figure 2:**
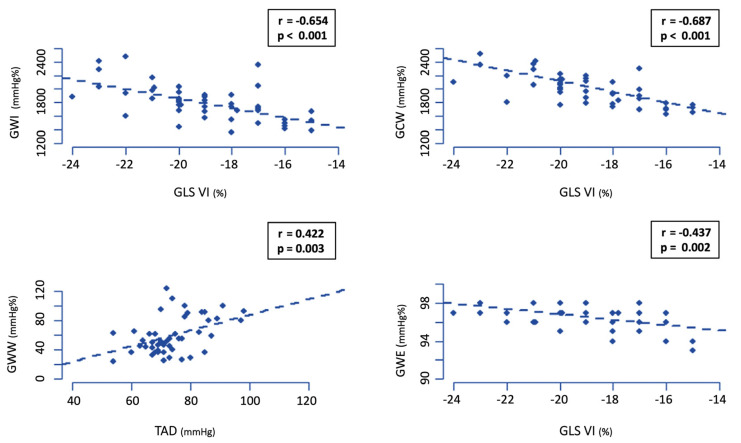
Main relations between global work index (GWI), global constructive work (GCW), global work waste (GWW), and global work efficiency (GWE).

For the professional football players, the best correlations for GWI, GCW, GWW, and GWE are shown in Figure 2. The remaining significant correlations for these four parameters in the players and controls are shown in Supplementary Table S3. Irrespective of the MW variable, the parameters that correlated best across all the populations a=were SBP, DBP, and GLS. In the control group, the variables with the highest correlation values were E, medial E/E’, tricuspid DTI systolic velocity, and GLS. Among the professional players, there were more variables of advanced echocardiography with significant correlations: LVESV, RVEDV, and RVESV.

Significant differences in GLS (*p* = 0.01) and GWE (*p* = 0.04) were observed as a function of the septal thicknesses of the athletes. Those with IVSTd < 10 mm presented higher GLS values (median: −20.00; IQR: −22.00, −18.00) and higher GWE values (median: 97.00; IQR: 96.00, 97.00) than those in whom IVSTd = 10–13 mm (median: −18.00; IQR, −19.00, −16.25) (median: 95.50; IQR: 93.00, 97.00).

**Figure 3:**
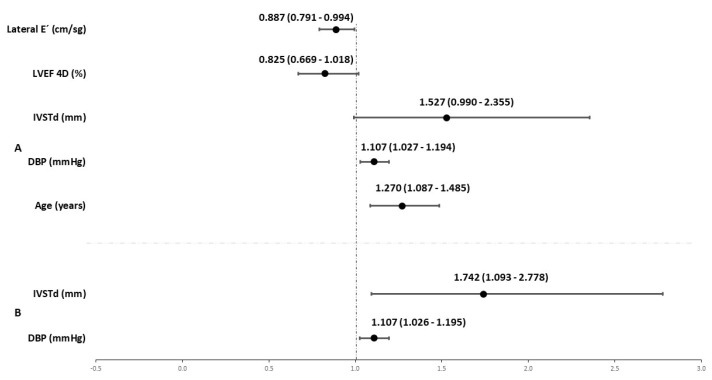
Physiological and anatomical parameters are associated with regurgitation (model **A**) and two or more alterations (model **B**). The *p*-values of model **A**: DBP (*p* = 0.009); IVSTd (*p* = 0.020). The *p*-values of model **B**: Age (*p* = 0.003); DBP (*p* = 0.008); IVSTd (*p* = 0.056); LVEF 4D (*p* = 0.073); lateral E´ (*p* = 0.039).

The proportion of valvular regurgitations (Supplementary Table S4) was unbalanced between the professional players (51.0%) and the controls (6.3%). The association analyses reported a higher prevalence of tricuspid and pulmonary regurgitations in the professional football players (*p* < 0.001). This imbalance was also observed in the number of cardiac alterations, which were present in 83.7% of the football players versus 14.6% of the controls. The significant or near-significant results of the binary logistic regression models are shown in Figure 3, according to which the variable with the most promising findings is IVSTd (mm). In the professional players, a one-unit increase in this parameter increased the probability of presenting a regurgitation by 1.742 times (*p* = 0.020) and of presenting more than one alteration by 1.527 times (*p* = 0.056).

## 4. Discussion

Standard echocardiography has an essential role in assessing the characteristics of the athletes´ hearts, differentiating physiological and pathological LV remodeling [18]. The hemodynamic conditions, specifically changes in cardiac output and peripheral vascular resistance, vary significantly across sporting disciplines. Our main findings were that the MW index can be a helpful method compared to conventional echocardiogram to address differences in athletes who present physiological remodeling of the heart, which was previously associated with better exercise capacity and the preservation of contractile reserves during physical effort [19]. In this sense, football involves some element of stress, as well as isotonic and isometric physiology [20], and generally presents less demand in terms of oxygen-transport capacity than long-distance running or cycling. Nevertheless, football has been shown to induce remodeling similar to sports with a predominant endurance component [21].

The 2DSTE analysis showed a mild but statistically significant reduction in LV GLS in the professional football players, who displayed lower significative GCW than the control group and a tendency toward lower GWI values, associated with normal biplane LVEF. These findings suggest that professional football players have greater anatomical cardiac remodeling, less deformation, and lower myocardial work indices due to their adaptation to more intensive training.

It is remarkable that when using normality-reference values of myocardial work [17], the professional players presented 94.7% lower values of GWW, which represented the work that was unproductive in relation to the LV ejection fraction, and 89.5% higher values of GWE, which may be explained by the players’ adaptation to intense exercise. As expected due to the results of previous studies [22], the GWI showed a good correlation with the SBP and global longitudinal strain, the GWW was significantly correlated with the DBP, and the GWE showed a good correlation with the global longitudinal strain. However, we did not find a strong correlation between the MW index and LV size with advanced 2DE/4D parameters of LV systolic function.

Although we could not find a significant difference in the GLS and BP between both groups, and even found a higher LV-mass index among the professional football players, this could have been determined by the intraventricular thickness differences between some of the LV segments. To this end, we performed an exploratory analysis, identifying an interesting finding: the professional players with an IVSTd of 10–13 mm presented poorer GLS (i.e., lower) and GWE (i.e., lower) values than their counterparts with left-ventricular septal thicknesses of less than 10 mm. Indeed, it is well-known that some trained individuals have an increased septal thickness [23], which may lead to lower strain and MW-derived values, as was found in our work. In this regard, we saw left- and right-ventricle dilation and increased LV-mass indices without an increase in absolute mean LV-wall thickness (mean values: RWT 0.32, IVSTd 8.08 mm). Nevertheless, the measures were within the established reference values. Furthermore, these findings are consistent with other published data on cohorts of male football players [24].

Additionally, we observed an association between professional football practice and mild pulmonary and tricuspid insufficiency (*p* < 0.001). Nevertheless, the regurgitation was generally mild and not clinically significant. Similarly, in a previous study, an increased prevalence of regurgitant flow was noted [25] in ultra-endurance athletes compared to sedentary control subjects, with a higher prevalence in those with greater training mileage. The regurgitation mechanism in structurally normal valves is unknown, but the authors suggested that this is an adaptative cardiac feature to strenuous exercise.

From a practical point of view and concerning daily practice, the measurement of myocardial work could provide additional information regarding functional cardiac adaptations, especially in athletes who often experience variable BP and loading conditions throughout their seasonal training. This would help to distinguish physiological changes derived from vigorous training from those that could be pathological or in the so-called grey zone without increasing the time devoted to technical investigations.

This study has some limitations, namely: (i) the small sample size and statistical power, which reduced the likelihood of reaching definitive conclusions; (ii) the lack of a myocardiopathy cohort, which might have allowed a better differentiation between the physiological and pathological adaptations; and (iii) a lack of cardiopulmonary test values, which might have helped form a better understanding of the findings of this work. However, our study has a strength, in that it is the first study to present an analysis of MW in a cohort of professional football players with identical degrees of training and, therefore, a similar cardiovascular demand, during the same period. Nevertheless, further investigations are warranted.

## 5. Conclusions

The MW is a novel index of LV systolic function that provides new insights into athletes’ myocardial properties. However, further evidence is needed to refine MW diagnostic performance and make it part of the standard diagnostic workup and differential diagnosis of other pathological conditions.

## Figures and Tables

**Table 1:** Demographic and echocardiographic characteristics of professional football players and controls.

	All Population (N = 97)		Professional Football Players (N = 49)		Controls (N = 48)		*p*-Value
	Mean	SD	Mean	SD	Mean	SD	
Age (years)	30.48	7.20	26.98	4.85	34.06	7.49	<0.001
Height (m)	1.76	0.10	1.77	0.09	1.74	0.10	0.063
Weight (Kg)	72.80	13.42	71.85	9.34	73.77	16.64	0.483
BMI (Kg/m^2^)	23.53	3.39	22.76	1.37	24.32	4.51	0.302
SBP (mmHg)	120.01	14.46	117.39	13.25	122.69	15.27	0.024
DBP (mmHg)	75.38	10.96	73.71	10.05	77.08	11.67	0.131
HR (lpm)	59.31	10.25	57.08	8.91	66.93	11.19	<0.001
Standard echocardiographic parameters							
LVIDd (mm)	49.65	4.40	51.29	3.27	47.98	4.80	<0.001
IVSTd (mm)	7.79	1.60	8.08	1.51	7.50	1.65	0.066
PWTd (mm)	7.29	1.20	7.55	1.19	7.02	1.16	0.023
RWT	0.31	0.06	0.32	0.06	0.31	0.06	0.748
GLS VI (%)	−19.63	2.72	−19.03	2.34	-20.23	2.96	0.030
LV mass indexed (g/m^2^)	62.65	12.61	65.02	14.07	57.24	5.72	0.011
LVEDV 2D (mL)	111.16	30.85	126.45	20.36	95.56	32.06	<0.001
LVESV 2D (mL)	42.79	16.16	48.84	13.91	36.63	16.09	<0.001
LVEF 2D (mL)	63.98	6.03	63.84	6.19	64.13	5.92	0.815
E (cm/s)	64.68	27.69	63.96	28.03	65.56	27.61	0.561
EA (ratio)	1.79	0.53	1.96	0.51	1.57	0.47	<0.001
Lateral E’ (cm/s)	13.94	5.03	13.52	5.98	14.52	3.33	0.919
Medial E’ (cm/s)	12.34	2.65	12.61	2.97	11.94	2.10	0.251
Lateral E/É	5.78	2.51	5.63	2.89	6.12	1.28	0.008
Medial E/É	5.72	1.48	5.50	1.50	6.09	1.40	0.109
TAPSE (mm)	23.75	3.37	24.44	3.41	23.00	3.19	0.019
Tricuspid DTI Systolic Velocity (cm/s)	13.39	1.57	13.31	1.62	13.48	1.53	0.789
Shortening fraction (%)	53.36	6.43	53.06	6.60	54.57	5.82	0.490
LAV (ml/m^2^)	24.56	8.62	24.30	7.58	25.01	10.33	0.973
Advanced echocardiographic parameters							
LVEDV 4D (mL)	138.55	31.83	148.74	25.93	118.17	33.23	<0.001
LVESV 4D (mL)	55.09	13.48	58.07	12.12	49.13	14.35	0.008
LVEF 4D (%)	60.65	3.59	59.91	3.18	62.13	3.95	0.014
LVSV 4D (mL)	85.14	18.87	89.73	16.29	74.53	20.56	0.003
LVCO 4D (L/m^2^)	4.55	0.92	4.56	0.85	4.55	1.09	0.817
RVEDV 4D (mL)	105.30	27.62	111.95	23.05	79.27	29.58	<0.001
RVESV 4D (mL)	45.96	12.64	47.88	11.16	37.70	15.74	0.020
RVEF 4D (%)	57.58	4.27	57.65	4.35	57.28	4.16	0.801
RVSV 4D (mL)	61.98	19.23	65.37	18.00	45.78	17.34	0.009
Myocardial Work							
GWI (mmHg%)	1840.33	269.58	1792.75	267.69	1887.92	265.73	0.084
GCW (mmHg%)	2133.03	307.73	2040.42	291.62	2225.65	298.08	0.003
GWW (mmHg%)	65.66	32.17	59.60	24.77	71.71	37.47	0.143
GWE (mmHg%)	96.22	1.75	96.56	1.30	95.88	2.06	0.088

Abbreviations: BMI: body-mass index, SBP: systolic blood pressure, DBP: diastolic blood pressure, HR: heart rate, LVIDd: left-ventricular internal diastolic diameter, IVSTd: diastolic interventricular septal thickness, PWT: diastolic posterior-wall thickness, RWT: relative wall thickness, LVEDV: left-ventricular end-diastolic volume, LVESV: left end-systolic volume, LVEF: left ventricular ejection fraction, LVCO: left-ventricular cardiac output, LVSV: left-ventricular stroke volume, LAV: left atrial volume, GLS: global longitudinal strain, RVEDV: right-ventricular end-diastolic volume, RVESV: right-ventricular end-systolic volume, RVEF: right-ventricular ejection fraction, RVSV: right-ventricular stroke volume, GWI: global myocardial work index, GCW: global constructive work, GWE: global myocardial work efficiency, GWW: global wasted work.

## Data Availability

All data are kept by the investigators of the study, and can be provided upon request.

## Supplementary Materials

The following supporting information can be downloaded at: https://www.mdpi.com/article/10.3390/jcm12093059/s1, Table S1: Demographic and echocardiographic characteristics depending on the position in the field. Table S2. Demographic and echocardiographic characteristics depending on the number of cardiac alterations. Table S3. Correlation matrix of the variables referring to MW and those referring to standard echocardiographic and advanced echocardiographic in amateur athletes and professional soccer players. Table S4. Prevalence of the different types of regurgitation in professional football players and controls.

## Abbreviations

2D-STE 2D: speckle-tracking echocardiography; BP: blood pressure; DBP: diastolic blood pressure; ECG: electrocardiogram; GLS: global longitudinal strain; GCW: myocardial constructive work; GWE: myocardial work efficiency; GWI: global work index; GWW: myocardial wasted work; IVSTd: diastolic interventricular septal thickness; LA: left atrial, LV: left-ventricular; LVEDV: left-ventricular end-diastolic volume; LVESV: left-ventricular end-systolic volume; LVEF: left-ventricular ejection fraction; LVIDd: left-ventricular-internal-dimension diastole; LVMI: left-ventricular-mass index; MW: myocardial work; PPS: pre-participation screening; PWTd: diastolic posterior-wall thickness; ROI: region of interest; RVEDV: right-ventricular end-diastolic volume; RVESV: right-ventricular end-systolic volume; RVEF: right-ventricular ejection fraction; RVSV: right-ventricular stroke volume; RWT: relative wall thickness; SBP: systolic blood pressure; TD: tissue Doppler.
