# Supplementary material for: Myocardial Work Index in Professional Football Players: A Novel Method for Assessment of Cardiac Adaptation
Source: J Clin Med. 2023 Apr 23;12(9):3059. doi: 10.3390/jcm12093059 (PMC10179020; doi:10.3390/jcm12093059)
Supplement: Supplementary file 1 [file jcm-12-03059-s001.zip › jcm-2201718-supplementary.pdf]

Supplementary Table S1. Demographic and echocardiographic characteristics depending on the position in the field.

	Goalkeeper and Defense (N = 19)		Midfielder (N = 16)		Striker (N = 13)		p-value
	Mean	SD	Mean	SD	Mean	SD	
Age (years)	27.05	4.02	28.00	4.32	24.69	5.22	0.145
Height (m)	1.80	0.09	1.76	0.08	1.76	0.11	0.466
Weight (Kg)	73.44	11.06	70.33	7.28	71.61	9.58	0.739
BMI (Kg/m^2)	22.59	1.50	22.74	1.26	23.03	1.43	0.725
SBP (mmHg)	118.89	11.64	111.25	11.01	118.77	8.33	0.037
DBP (mmHg)	74.37	8.18	71.88	10.40	73.23	10.83	0.715
HR (bpm)	57.06	7.84	56.44	11.18	58.31	7.95	0.761
**Standard echocardiographic parameters**							
LVIDd (mm)	51.37	2.91	51.06	3.57	51.54	3.71	0.890
IVSTd (mm)	8.00	1.33	8.25	1.18	7.92	2.14	0.654
PWTd (mm)	7.63	1.12	7.50	1.41	7.54	1.13	0.988
RWT	0.31	0.05	0.33	0.06	0.31	0.09	0.805
GLS VI (%)	-19.14	2.34	-19.31	2.38	-18.67	2.46	0.754
LV mass indexed (g/m^2)	69.43	14.79	60.47	8.66	65.58	17.67	0.211
LVEDV 2D (ml)	123.95	24.46	125.88	19.46	130.23	16.02	0.743
LVESV 2D (ml)	49.74	14.68	43.94	10.77	51.38	14.12	0.320
LVEF 2D (%)	63.68	5.94	65.44	6.78	62.62	5.80	0.556
E (cm/s)	61.72	29.77	62.42	28.47	70.70	27.11	0.502
EA (ratio)	1.95	0.55	1.93	0.39	2.05	0.63	0.755
Lateral E´ (cm/s)	12.08	6.12	15.11	5.81	13.83	5.87	0.215
Medial E´(cm/s)	12.38	2.71	13.81	2.76	11.64	3.17	0.193
Lateral E/É	6.05	3.35	4.90	2.54	5.94	2.58	0.143
Medial E/É	5.67	1.83	5.18	1.09	5.57	1.58	0.863
TAPSE (mm)	24.18	3.72	25.25	3.86	23.92	2.40	0.557
Tricuspid DTI Systolic Velocity (cm/s)	13.61	1.88	13.12	1.27	13.01	1.89	0.687
Shortening fraction (%)	52.00	7.62	52.38	6.13	55.58	5.45	0.368
LAV (ml/m^2)	23.81	7.84	23.47	6.73	26.39	8.66	0.491
**Advanced echocardiographic parameters**							
LVEDV 4D (ml)	150.44	24.29	146.88	29.63	148.67	25.14	0.944

LVESV 4D (ml)	59.33	10.84	55.88	13.25	59.08	12.99	0.657
LVEF 4D (%)	60.15	3.21	60.75	3.36	58.42	2.57	0.122
LVSV 4D (ml)	94.38	12.45	89.13	18.31	84.34	17.48	0.215
LVCO 4D (l/m^2)	4.69	1.01	4.41	0.81	4.55	0.68	0.561
RVEDV 4D (ml)	116.56	27.61	109.75	19.68	108.45	21.25	0.648
RVESV 4D (ml)	49.88	11.90	47.38	11.18	45.73	10.56	0.641
RVEF 4D (%)	58.19	4.71	56.88	4.71	58.05	3.40	0.753
RVSV 4D (ml)	69.38	25.01	62.50	11.14	63.73	13.94	0.888
**Myocardial Work**							
GWI (mmHg%)	1806.16	198.88	1774.44	310.70	1748.67	278.63	0.645
GCW (mmHg%)	2043.37	230.59	2038.13	328.49	1985.42	297.63	0.569
GWW (mmHg%)	62.11	24.84	59.75	24.66	53.67	26.28	0.457
GWE (mmHg%)	96.42	1.22	96.63	1.36	96.67	1.50	0.506

Abbreviations. BMI: Body Max Index, SBP: Systolic Blood Pressure, DBP: Diastolic Blood Pressure, HR: Heart Rate, LVIDd: Left ventricular internal diastolic diameter, IVSTd: diastolic interventricular septal thickness, PWT: diastolic posterior wall thickness, RWT: relative Wall thickness, LVEDV: Left ventricular end-diastolic volume, LVESV: Left end-systolic volume, LVEF: Left ventricular ejection fraction, LVCO: Left ventricular cardiac output, LVSV: Left ventricular stroke volume, LAV: Left atrial volume, GLS: global longitudinal strain, RVEDV: Right ventricular end-diastolic volume, RVESV: Right ventricular end-systolic volume, RVSV: Right ventricular stroke volume, GWI: Global myocardial work index, GCW: Global constructive work, GWE: Global Myocardial work efficiency, GWW: Global Wasted work.

Supplementary Table S2. Demographic and echocardiographic characteristics depending on the number of cardiac alterations.

	Without alterations (N = 8)		1 alteration (N = 21)		2 or more alterations (N = 20)		p-value
	Mean	SD	Mean	SD	Mean	SD	
Age (years)	24.88	4.52	25.19	4.18	29.70	4.50	0.004
Height (m)	1.72	0.13	1.77	0.08	1.80	0.08	0.321
Weight (Kg)	67.31	8.92	71.87	9.48	73.64	9.20	0.489
BMI (Kg/m^2)	22.79	1.78	22.74	1.28	22.76	1.35	0.676
SBP (mmHg)	108.38	9.16	117.76	11.08	120.60	15.43	0.497
DBP (mmHg)	64.13	4.91	72.62	8.35	78.70	10.32	0.151
HR (bpm)	54.00	9.86	57.75	10.54	57.65	6.68	0.892
**Standard echocardiographic parameters**							
LVIDd (mm)	51.50	3.93	51.33	3.37	51.15	3.05	0.990
IVSTd (mm)	7.25	1.16	7.90	1.37	8.60	1.64	0.150
PWTd (mm)	7.88	1.55	7.48	1.17	7.50	1.10	0.967
RWT	0.28	0.05	0.31	0.07	0.34	0.06	0.192
GLS VI (%)	-18.88	2.17	-18.48	2.38	-19.72	2.31	0.079
LV mass indexed (g/m^2)	65.40	10.26	64.94	12.75	65.00	16.49	0.485
LVEDV 2D (ml)	125.13	23.90	130.95	15.85	122.25	23.04	0.611
LVESV 2D (ml)	42.63	11.81	51.14	13.42	48.90	15.03	0.629
LVEF 2D (%)	65.25	7.48	63.10	5.63	64.05	6.42	0.565
E (cm/s)	63.10	28.51	74.50	15.27	53.23	34.65	0.070
EA (ratio)	2.15	0.43	1.91	0.49	1.94	0.56	0.910
Lateral E´ (cm/s)	14.89	6.39	15.19	4.29	11.14	6.79	0.065
Medial E´(cm/s)	11.88	1.46	13.14	2.84	12.42	3.55	0.382
Lateral E/É	4.61	1.18	5.35	2.30	6.33	3.75	0.809
Medial E/É	5.37	1.05	5.20	1.12	5.79	1.87	0.371
TAPSE (mm)	25.50	4.41	23.07	3.11	25.45	2.91	0.013
Tricuspid DTI Systolic Velocity (cm/s)	12.82	1.10	13.62	1.39	13.13	2.17	0.709
Shortening fraction (%)	54.67	5.51	54.23	5.88	50.89	7.61	0.156
LAV (ml/m^2)	28.84	10.04	22.31	6.55	24.71	7.24	0.221
**Advanced echocardiographic parameters**							
LVEDV 4D (ml)	150.57	39.58	147.80	23.62	149.05	23.82	0.613

LVESV 4D (ml)	57.29	16.35	57.45	11.55	59.00	11.64	0.527
LVEF 4D (%)	62.14	3.29	60.10	3.14	58.88	2.86	0.193
LVSV 4D (ml)	89.71	28.36	89.63	14.30	89.84	13.07	0.648
LVCO 4D (l/m^2)	4.36	0.94	4.68	1.01	4.50	0.65	0.955
RVEDV 4D (ml)	106.29	25.99	104.05	21.74	124.31	18.91	0.007
RVESV 4D (ml)	46.00	11.87	44.55	12.02	52.88	8.16	0.010
RVEF 4D (%)	56.24	3.32	58.29	4.63	57.51	4.48	0.518
RVSV 4D (ml)	60.29	15.29	60.15	13.43	74.13	21.33	0.032
**Myocardial Work**							
GWI (mmHg%)	1679.75	220.65	1746.19	266.75	1891.79	265.60	0.148
GCW (mmHg%)	1910.25	214.18	1991.52	278.02	2149.26	309.05	0.088
GWW (mmHg%)	45.25	9.72	67.14	27.59	57.32	23.57	0.074
GWE (mmHg%)	97.13	0.35	96.19	1.54	96.74	1.19	0.297

Abbreviations. BMI: Body Max Index, SBP: Systolic Blood Pressure, DBP: Diastolic Blood Pressure, HR: Heart Rate, LVIDd: Left ventricular internal diastolic diameter, IVSTd: diastolic interventricular septal thickness, PWT: diastolic posterior wall thickness, RWT: relative Wall thickness, LVEDV: Left ventricular end-diastolic volume, LVESV: Left end-systolic volume, LVEF: Left ventricular ejection fraction, LVCO: Left ventricular cardiac output, LVSV: Left ventricular stroke volume, LAV: Left atrial volume, GLS: global longitudinal strain, RVEDV: Right ventricular end-diastolic volume , RVESV: Right ventricular end-systolic volume, RVSV: Right ventricular stroke volume, GWI: Global myocardial work index, GCW: Global constructive work, GWE: Global Myocardial work efficiency, GWW: Global Wasted work.

Supplementary Table S3. Correlation matrix of the variables referring to MW and those referring to standard echocardiographic and advanced echocardiographic in amateur athletes and professional soccer players.

**PROFESSIONAL FOOTBALL PLAYERS**								
Age (years)	0.205	0.163	0.209	0.155	0.285	0.049	-0.223	0.128
SBP (mmHg)	0.503	0.000	0.467	0.001	0.205	0.162	-0.190	0.196
DBP (mmHg)	0.208	0.156	0.271	0.063	0.422	0.003	-0.314	0.030
HR (bpm)	-0.258	0.080	-0.195	0.189	0.155	0.299	-0.182	0.221
**Standard echocardiographic parameters**								
LVESV 2D (ml)	-0.078	0.600	-0.050	0.738	0.277	0.057	-0.281	0.053
LVEF 2D (%)	0.136	0.356	0.152	0.303	-0.226	0.122	0.275	0.058
EA	0.142	0.345	0.131	0.386	-0.191	0.203	0.300	0.043
TAPSE (mm)	0.352	0.014	0.328	0.023	-0.187	0.203	0.276	0.058
Shortening fraction (%)	-0.305	0.047	-0.326	0.033	0.031	0.845	0.028	0.857
LAV (ml/m2)	0.381	0.009	0.370	0.011	-0.279	0.061	0.345	0.019
GLS VI (%)	-0.654	0.000	-0.687	0.000	0.249	0.088	-0.437	0.002
**Advanced echocardiographic parameters**								
LVSV 4D (ml)	0.293	0.056	0.214	0.169	-0.100	0.524	0.142	0.364
RVEDV 4D (ml)	0.401	0.008	0.466	0.002	-0.094	0.549	0.159	0.307
RVESV 4D (ml)	0.366	0.016	0.453	0.002	-0.035	0.824	0.111	0.479
RVSV 4D (ml)	0.347	0.023	0.365	0.016	-0.118	0.451	0.174	0.265
**CONTROLS**								
	**GWI**		**GCW**		**GWW**		**GWE**	
	Coefficient	p-value	Coefficient	p-value	Coefficient	p-value	Coefficient	p-value
BMI (Kg/m^2)	0.270	0.064	0.173	0.239	0.154	0.297	0.007	0.962
SBP (mmHg)	0.259	0.076	0.215	0.142	0.412	0.004	-0.254	0.082
DBP (mmHg)	0.164	0.264	0.134	0.365	0.394	0.006	-0.264	0.069
HR (bpm)	0.001	0.997	0.059	0.840	0.280	0.332	-0.204	0.485
**Standard echocardiographic parameters**								
RWT	-0.003	0.983	-0.050	0.733	0.223	0.128	-0.253	0.083
E (cm/sg)	0.352	0.028	0.325	0.044	-0.013	0.936	0.194	0.236
Medial E/É	0.369	0.063	0.333	0.096	0.104	0.614	0.069	0.739
Tricuspid DTI Systolic Velocity (cm/sg)	0.405	0.055	0.309	0.152	-0.326	0.129	0.431	0.040
GLS VI (%)	-0.595	0.000	-0.613	0.000	0.403	0.005	-0.534	0.000

Advanced echocardiographic parameters								
LVSV 4D (ml)	-0.295	0.172	-0.363	0.089	0.070	0.752	-0.087	0.694

Abbreviations. BMI: Body Max Index, SBP: Systolic Blood Pressure, DBP: Diastolic Blood Pressure, HR: Heart Rate, LVIDd: Left ventricular internal diastolic diameter, IVSTd: diastolic interventricular septal thickness, PWT: diastolic posterior wall thickness, RWT: relative Wall thickness, LVEDV: Left ventricular end-diastolic volume, LVESV: Left end-systolic volume, LVEF: Left ventricular ejection fraction, LVCO: Left ventricular cardiac output, LVSV: Left ventricular stroke volume, LAV: Left atrial volume, GLS: global longitudinal strain, RVEDV: Right ventricular end-diastolic volume , RVESV: Right ventricular end-systolic volume, GWI: Global myocardial work index, GCW: Global constructive work, GWE: Global Myocardial work efficiency, GWW: Global Wasted work.

Supplementary Table S4. Prevalence of the different types of regurgitation in professional football players and controls.

	**Mitral Regurgitation**		**Tricuspid Regurgitation**		**Pulmonary Regurgitation**		**Aortic Regurgitation**		**Any Regurgitation**	
	N	%	N	%	N	%	N	%	N	%
**Professional Football Players**	8	16.3	17	34.7	13	26.5	1	2.0	25	51.0
**Controls**	3	6.3	0	0.0	0	0.0	0	0.0	3	6.3
**p-value**	0.118		<0.001		<0.001		0.320		<0.001	
